# Excretory/Secretory Proteome of Females and Males of the Hookworm *Ancylostoma ceylanicum*

**DOI:** 10.3390/pathogens12010095

**Published:** 2023-01-06

**Authors:** Samuel C. Uzoechi, Bruce A. Rosa, Kumar Sachin Singh, Young-Jun Choi, Bethany K. Bracken, Paul J. Brindley, R. Reid Townsend, Robert Sprung, Bin Zhan, Maria-Elena Bottazzi, John M. Hawdon, Yide Wong, Alex Loukas, Sergej Djuranovic, Makedonka Mitreva

**Affiliations:** 1 Division of Infectious Diseases, Department of Medicine, Washington University School of Medicine, St. Louis, MO 63110, USA; 2 Charles River Analytics, Inc., Cambridge, MA 02138, USA; 3 Department of Microbiology, Immunology & Tropical Medicine, Research Center for Neglected Diseases of Poverty, School of Medicine and Health Sciences, George Washington University, Washington, DC 20037, USA; 4 Division of Endocrinology, Metabolism and Lipid Research, Department of Medicine, Washington University School of Medicine, St. Louis, MO 63110, USA; 5 Department of Pediatric Tropical Medicine, National School of Tropical Medicine, Baylor College of Medicine, Houston, TX 77030, USA; 6 Centre for Molecular Therapeutics, Australian Institute of Tropical Health and Medicine, James Cook University, Cairns 4878, Australia; 7 Department of Cell Biology and Physiology, Internal Medicine, Washington University School of Medicine, St. Louis, MO 63110, USA

**Keywords:** hookworm, *Ancylostoma ceylanicum*, excretory/secretory, ESP, proteomics

## Abstract

The dynamic host-parasite mechanisms underlying hookworm infection establishment and maintenance in mammalian hosts remain poorly understood but are primarily mediated by hookworm’s excretory/secretory products (ESPs), which have a wide spectrum of biological functions. We used ultra-high performance mass spectrometry to comprehensively profile and compare female and male ESPs from the zoonotic human hookworm *Ancylostoma ceylanicum*, which is a natural parasite of dogs, cats, and humans. We improved the genome annotation, decreasing the number of protein-coding genes by 49% while improving completeness from 92 to 96%. Compared to the previous genome annotation, we detected 11% and 10% more spectra in female and male ESPs, respectively, using this improved version, identifying a total of 795 ESPs (70% in both sexes, with the remaining sex-specific). Using functional databases (KEGG, GO and Interpro), common and sex-specific enriched functions were identified. Comparisons with the exclusively human-infective hookworm *Necator americanus* identified species-specific and conserved ESPs. This is the first study identifying ESPs from female and male *A. ceylanicum*. The findings provide a deeper understanding of hookworm protein functions that assure long-term host survival and facilitate future engineering of transgenic hookworms and analysis of regulatory elements mediating the high-level expression of ESPs. Furthermore, the findings expand the list of potential vaccine and diagnostic targets and identify biologics that can be explored for anti-inflammatory potential.

## 1. Introduction

Parasitic infections caused by blood-feeding hookworms are an ongoing global health threat. Hookworms are nematode roundworms of the Ancylostomatidae family, a part of the Strongylida order [1]. Humans are infected with hookworms through ingestion and/or skin penetration of the infectious third-stage larvae (iL3). Worldwide, *Ancylostoma duodenale* and *Necator americanus* were believed to be the most prevalent hookworm species, but more recently, *Ancylostoma ceylanicum* appeared as an important human hookworm parasite in some countries [2]. More than 500 million people including children are affected [3], with the highest prevalence occurring in tropical regions of Asia followed by Sub-Saharan Africa and North and South America [4,5,6,7]. According to CDC, light hookworm infections may be asymptomatic and relatively benign in most cases, although individuals with chronic hookworm infection may experience fatigue, abdominal pain, diarrhea, loss of appetite, weight loss, and iron deficiency anemia [7]. In prevalent regions, children between 2–11 years old are more exposed to hookworm infection which retards physical and cognitive development and leads to anemia and malnutrition [8,9]. Hookworm infection is prevalent in resource-limited areas in most endemic regions with limited access to sanitation, and sewage treatment [10]. There is evidence suggesting that diseases concomitant with prolonged hookworm infection may be a predisposing risk factor to other tropical diseases including tuberculosis, HIV/AIDS, and malaria [11,12]. Total productivity losses as a result of global hookworm infections are estimated to be between $11 billion and $138 billion annually (depending on the calculation approach used) [13].

The life cycle of *A. ceylanicum* is straightforward, involving just a single mammalian host, including dogs, cats, and humans [14,15], with no intermediate host required. The adult female hookworms in the small intestine lay eggs that are passed out of the host body in the feces, and under favorable conditions, the eggs embryonate in soil and hatch to release the first-stage larva (L1). The larvae undergo a series of molts to reach the infective third stage (iL3) that can be ingested or penetrate the skin of the host [3]. Upon infection, the iL3 parasite moves to the lungs through the circulatory system. Once in the pulmonary vasculature, the iL3 break into the alveolar spaces and migrate up the trachea where they trigger mild irritation that causes the host to cough. Coughing moves the larvae to the mouth, and they are eventually swallowed, whereupon they enter the gastrointestinal tract, ultimately arriving in the duodenum, where they attach to the small intestine, and develop into adult blood-feeding male and female worms [3].

The adult stage of hookworms excretes and secretes a wide range of structurally and functionally distinct macromolecules called excretory/secretory products (ESPs), mostly comprised of arrays of proteins that are believed to interact with host tissues and stimulate parasite survival [16,17]. In addition to these proteins, ESPs include carbohydrates and lipids, and extracellular vesicles containing nucleic acids are constantly pumped from the parasite’s outer surfaces. Based on the wide spectrum of biological functions for ESPs, they are thought to be involved in critical host-parasite interactions that mediate parasite survival in the small intestine and contribute to hookworm infection [6]. Because of this, these macromolecules are strong potential vaccines and drug targets for eradicating hookworm infection, and drug targets for many inflammatory diseases [18]. Moreover, hookworm ESPs contain molecules with therapeutic potentials for treating a whole range of immune-mediated inflammatory diseases [19,20,21]. Published ESP studies are mostly focused on other hookworm species (*N. americanus* [6] and *A. caninum* [22]), with no emphasis on *A. ceylanicum*. By contrast, little is known about the roles of *A. ceylanicum* ESPs in host-parasite interactions and immunoregulation compared to various parasites that serve as model organisms for human infections. Here, we performed a mass spectrometry analysis of the secretome of cultured supernatant of female and male *A. ceylanicum*. About 790 total proteins were detected in both female and male ESPs. The aim was to profile the ES proteins in female and male *A. ceylanicum* to identify common patterns and sex-specific proteins. These findings provide the first comprehensive proteomic characterization of ESPs of female and male *A. ceylanicum* and allowed us to refine the draft genome.

## 2. Materials and Methods

### 2.1. Ethics

The animal study was reviewed and approved by the Institutional Animal Care and Use Committee (IACUC) of Washington University in St. Louis (Protocol # 20-0323).

### 2.2. Hamster Gavage Procedure

Hamsters were gavaged using the appropriate technique and were carried out in accordance with the recommendations in the Guide for the Care and Use of Laboratory Animals of the National Institutes of Health (NIH). Freshly isolated infective *A. ceylanicum* L3 larvae were counted and distributed in individual Eppendorf tubes, and 80–100 L3 were resuspended per tube in 200 µL of PBS. A curved 18G needle was used to gavage the hamsters. Hamsters were manually restrained by grasping the loose skin at the scruff of the neck with the thumb and forefinger to immobilize the head and torso. Using the needle held next to the animal, the distance from the tip of the nose to the last rib on the left side was measured. Holding the animal with the nose pointing up, the gavage needle was entered into the mouth over the tongue and directed toward the esophagus on the left side of the throat, gently pressing the needle on the back of the mouth and allowing the animal to swallow the needle. The needle was slowly advanced to the measured distance into the stomach. Once the needle was properly positioned, the plunger was gently depressed to dispense ~200 µL of resuspended L3 larvae to the hamster. Once the entire inoculum was dispensed, the gavage needle was slowly removed, and the animal was returned to its cage. Gavaged hamsters were observed for any difficulty breathing or for bleeding from the mouth or nose for at least 5 min before returning them to the animal holding room.

### 2.3. Isolation of Adult Ancylostoma Ceylanicum

Twenty-one days post-infection (dpi) with 80 infective *A. ceylanicum* iL3, three Golden/Syrian hamsters were euthanized using the appropriate technique, equipment, and agents. Compressed CO_2_ gas in cylinders was used as a source of carbon dioxide, allowing for the controlled inflow of gas to the induction chamber. Hamsters to be euthanized were placed in a clean transparent chamber 100% CO_2_ was introduced at a fill rate of 70% of the chamber volume per minute, to achieve a balanced gas mixture with the existing air in the chamber to fulfill the objective of rapid unconsciousness with minimal distress to the animals. The expected time to unconsciousness was usually within 3–5 min. Each hamster was monitored for lack of respiration and faded eye color. CO_2_ was maintained for a minimum of 1 min after observing these signs to avoid unintended recovery upon exposure to natural CO_2_ concentrations. Upon completion of the procedure, death was confirmed by ascertaining cardiac and respiratory arrest and noting an animal’s fixed and dilated pupils. Following this euthanization procedure, the small intestines were removed and longitudinally cut open. The freshly recovered adult *A. ceylanicum* were gently washed three times in 1× PBS containing 5× Antibiotics/Antimycotics (Gibco, Waltham, MA, USA). Isolation of adult hookworms from hamsters was performed as previously described [23].

### 2.4. Excretory-Secretory Products (ESPs) Collection

Briefly, adult *A. ceylanicum* were collected in a 10 mm Petri Dish and placed on the inverted microscope stage with a 20× objective lens (Nikon Eclipse T_S_2). The freshly recovered adult *A. ceylanicum* were sorted with a nail brush under the microscope to group into females and males. Each group was cultured in three biological replicates at a density of 25 worms/well in 24 well plates. The worms were first cultured for 4 h in a humidified incubator at 37 °C, 5% CO_2_, Dulbecco’s Modification of Eagle’s Medium (DMEM) (Corning, Corning, NY, USA) supplemented with 2% Antibiotic-Antimycotic (Gibco) and 10% Fetal Bovine Serum (Sigma, St. Louis, MI, USA) [24]. The supernatant was discarded, and the worms were gently rinsed twice in 1× PBS containing 5× Antibiotics/Antimycotics. Afterward, the worms were subcultured in 2 mL serum-free DMEM supplemented with 2% Antibiotic-Antimycotic at 37 °C, and 5% CO_2_ [24]. Every 24 h, over 3 days, at least 1.8 mL of culture supernatant was aspirated from each well with minimal disturbance to the hookworms. Each well was replaced with fresh serum-free medium, and plates were transferred to the incubator, as described [6]. The drawn culture supernatant was centrifuged at 4000× *g* at 4 °C for 30 min and transferred (all but the last 1.6 mL) to a new centrifuge tube and stored at −80 °C. Using this method for further media changes prevented voided eggs and remaining hamster or dead worm tissue from contaminating the ESPs [6]. After 72 h, no significant alterations in motility or morphological integrity of the worms were detected, suggesting that the drawn media was comprised of ESPs from the normal physiological activity of healthy adult *A. ceylanicum*. However, it should be noted that these ESPs are from worms collected from a hamster host and cultured in media to facilitate the collection, but differences may exist in ESPs actually produced in a human intestine.

At the end of 72 h of media collection, the culture supernatant containing ESPs was gradually thawed in ice. To maximize the peptide yield, the three biological replicates were pooled into a 15 mL tube. Approximately, 14 mL of culture supernatant was recovered from 24 to 72 h of media collection per group. The crude ESPs were further cleaned up by centrifugation at 7000× *g* at 4 °C for 30 min before concentration and transferred (all but the last 13 mL) to a new tube. The culture media containing ESPs was concentrated using 20 mL Pall Macrosep Advance Centrifugal Devices with a defined 3000 Da molecular weight cutoff membrane. Briefly, 13 mL of culture supernatant was placed into the concentrator sample chamber and capped. The concentrator was then placed into the bucket rotor that contains a 20 mL tube. Concentration by 3K Pall membrane was performed at 5000× *g* at 4 °C until the retention volume reaches 0.5 mL.

### 2.5. Quantification of Protein Concentration

For total proteomic profiling of adult female and male *A. ceylanicum* proteins, daily ESPs collections of each sex were pooled and quantified. ESPs concentrations were quantified using a Pierce Coomassie Plus Bradford Protein Assay (Thermo Scientific, Waltham, MA, USA). A BSA standard at 1 mg/mL was dissolved in DMEM (Corning) supplemented with 2% Antibiotic-Antimycotic (Gibco). Separately, 5 µL of each standard and ESPs sample were then added to 395 µL of 1× Bradford dye in a 96-well plate. Following 5 min of incubation in the dark at room temperature, the optical density (OD) was measured at 595 nm using Eon Microplate Spectrophotometer (Biotek Instruments, Winooski, VT, USA). A standard curve was generated by plotting the graph of OD (y-axis, nm) versus protein concentration (x-axis, µg/mL). The protein concentrations of both female and male ESPs were then estimated based on linear extrapolation from the standard curve.

### 2.6. SDS-PAGE

Approximately 3 μg of *A. ceylanicum* adult ESPs was mixed with 2× concentrated Laemmli-SDS buffer (Bio-Rad) with 5% 2-Mercaptoethanol (Sigma-Aldrich). The sample was heated at 95 °C for 5 min and 50 µL of protein sample was loaded into the wells of 12% Mini-PROTEAN TGX gel (Bio-Rad). The precision protein standard (Bio-Rad) was loaded into the first well at 10 µL without dilution with an SDS sample buffer. Electrophoresis was run for 50 min at 150 V using 1× Tris/Glycine buffer (Bio-Rad), [6]. The gel was fixed in (40% ethanol and 10% acetic acid), washed with distilled water, and stained with QC Colloidal Coomassie Stain (Bio-Rad). Gels were imaged in Azure Imaging Systems (C600, Azure Biosystems, Dublin, CA, USA) under LED visible fluorescence detection mode (data not shown).

### 2.7. Sample Preparation for Mass Spectrometry

Concentrated media samples (300 µL of control media, 150 µL of male/female worm media) were precipitated in −20 °C acetone (1 part of the sample to 4 parts cold acetone (vol/vol)) overnight followed by centrifugation at 20,000 rcf at for 10 min. Supernatants were removed and media protein pellets were solubilized in 20 µL lysis buffer (8 M urea (Sigma, Cat. No. U4884-500g), 100 mM Tris-HCl buffer (Fisher, Cat. No. BP-152-1), pH 8.0). Samples were reduced with 5 mM DTT (Pierce, Cat. No. A39255) for 1 h at 37 °C. The reduced Cys residues were alkylated with 10 mM iodoacetamide (Pierce, Cat. No. A39271) for 45 min at room temperature in the dark. Samples were diluted 1:4 with 50 mM Tris-HCl, pH 8.0, and proteins were digested with LysC (Wako Chemicals, Cat. No. 129-02541) (1 µg) for 2 h at 25 °C in a ThermoMixer (Eppendorf) with shaking at 1000 rpm. Trypsin (Promega, Cat. No. V511C) (1 µg) was added and digestion was performed in the ThermoMixer (100 rpm) overnight at 25 °C. The digestion was quenched by the addition of concentrated formic acid (Fluka) to a final concentration of 1% (vol/vol). Peptides were desalted using stage tips [25] and eluted in 50% (vol/vol) MeCN containing 0.1% (vol/vol) FA. An aliquot (1%) was removed for quantification using the Pierce Quantitative Fluorometric Peptide Assay kit (ThermoFisher, Cat. No. 23290). The remaining peptides were transferred to autosampler vials (Sun-Sri, Cat. No. 200046), dried, and stored at −80 °C.

### 2.8. UPLC-MS

The samples were analyzed using ultra-high-performance mass spectrometry [26] using a hybrid quadrupole Orbitrap LC-MS System, Q-Exactive™ PLUS interfaced to an EASY-nanoLC 1000). A 75 μm i.d. × 50 cm Acclaim PepMap 100 C18 RSLC column (Thermo Scientific™) was equilibrated with 100% solvent A (1%FA) on the nano-LC for a total of 11 μL at 700 bar pressure. Samples in 1% FA (vol/vol) were loaded at a constant pressure of 700 bar. Peptide chromatography was initiated with mobile phase A (1% FA) containing 2% solvent B (100%ACN, 1% FA) for 5 min, then increased to 20% B over 100 min, to 32% B over 20 min, to 95% B over 1 min and held at 95% B for 19 min, with a flow rate of 250 nl/min. Data were acquired in data-dependent mode. Full-scan mass spectra were acquired with the Orbitrap mass analyzer using a scan range of *m/z* = 325 to 1800 and a mass resolving power set to 70,000. Ten data-dependent high-energy collisional dissociations were performed with a mass resolving power at 17,500, a fixed lower value of *m/z* 110, an isolation width of 2 Da, and a normalized collision energy setting of 27. The maximum injection time was 60 ms for parent-ion analysis and production analysis. Ions that were selected for MS/MS were dynamically excluded for 30 sec. The automatic gain control was set at a target value of 1 × 10^−6^ ions for full MS scans and 1 × 10^−5^ ions for MS2. Peptide ions with charge states of one were excluded from MS2 acquisition. The MS2 spectra from peptides with +2, +3, and +4 charge states were analyzed using Mascot software [27] (Matrix Science, London, UK; version 2.5.1). The mascot was searched against the improved *A. ceylanicum* proteome (22,946 entries, described above), using trypsin enzyme specificity, with a maximum of 2 missed cleavages allowed/peptide. The searches were performed with a fragment ion mass tolerance of 20 ppm and a parent ion tolerance of 20 ppm. Carbamidomethylation of cysteine was specified as a fixed modification. Deamidation of asparagine, deamidation of glutamine, acetylation of protein N-terminus, and oxidation of methionine were entered as variable modifications. The mass spectrometry proteomics data have been deposited to the ProteomeXchange Consortium (http://proteomecentral.proteomexchange.org, accessed on 1 November 2022) via the iProX partner repository [28] with the dataset identifier PXD037410, under project ID IPX0005214000. The accessions for each sample are IPX0005214001 (Adult_Female_ESPs), IPX0005214002 (Adult_Male_ESPs), and IPX0005214003 (Media-only control). The dataset on iProX includes the .sf3-format Scaffold output file (as described below).

### 2.9. Genome Annotation Improvement and Peptide Identification

A previously published *A. ceylanicum* genome assembly [29] was re-annotated using the MAKER annotation pipeline v3.1.3 [30] to improve the quality of protein-coding gene models. Repetitive elements were softmasked with RepeatMasker v4.1.2 using a species-specific repeat library created by RepeatModeler v2.0.2 [31]. PacBio cDNA sequencing [32] and Illumina RNA-seq (PRJNA72583) data were assembled and used as transcript evidence. Specifically, Clontech SMARTer cDNA synthesis primer sequences were removed from the PacBio CCS reads using lima v2.2.0 and cutadapt v3.4. After poly-A trimming, these reads were aligned to the reference genome using minimap2 [33] following a two-pass approach [34], and subsequently assembled using StringTie2 v2.1.6 (long-read mode) [35]. Illumina RNA-seq data were adapter/quality trimmed using Trimmomatic v0.39 [36], and reference-aligned using HISAT2 v2.2.0 [37] with the dta option and assembled using StringTie2 v2.1.6. Protein sequences from WormBase ParaSite Version 15 (October 2020) [38] (*Ancylostoma caninum* PRJNA72585, *Caenorhabditis elegans* PRJNA13758, *Haemonchus contortus* PRJEB506, *Mesorhabditis belari* PRJEB30104, *Micoletzkya japonica* PRJEB27334, *Necator americanus* PRJNA72135, *Nippostrongylus brasiliensis* PRJEB511, *Oscheius tipulae* PRJEB15512, *Pristionchus pacificus* PRJNA12644) were provided to MAKER as protein homology evidence. Gene predictions generated using BRAKER2 v2.1.6 [39] based on the Illumina RNA-seq alignments, together with the above-mentioned evidence data, were synthesized into gene annotations within the MAKER pipeline. Gene predictions without supporting evidence were excluded in the final annotation build, with the exception of those encoding Pfam domains, as detected by InterProScan v5.50 [40] in order to improve the annotation accuracy by balancing sensitivity and specificity [30,41]. The completeness of annotated gene set was assessed using BUSCO v4.1.4 with Nematoda-specific single-copy orthologs (OrthoDB v10) [42]. The newly annotated, improved gene models were used in subsequent proteomic analysis of ESPs, and are publicly available on Nematode.net [43,44]. Functional annotations, re-mapped life cycle RNA-seq read counts and relative gene expression levels (mRNA levels) [45], and proteomics results (described below) for each of the newly annotated genes are provided in Supplementary Table S1.

Scaffold (version Scaffold_5.1.0, Proteome Software Inc., Portland, OR) was used to validate MS/MS-based peptide and protein identifications. Peptide identifications were accepted if they could be established at greater than 7.0% probability to achieve an FDR less than 1.0% by the Scaffold Local FDR algorithm. Protein identifications were accepted if they could be established at greater than 95.0% probability and contained at least 2 identified peptides. Protein probabilities were assigned by the Protein Prophet algorithm [46]. Proteins that contained similar peptides and could not be differentiated based on MS/MS analysis alone were grouped to satisfy the principles of parsimony. Peptide-spectral match (PSM) counts for each peptide sequence are provided in Supplementary Table S2, and the raw scaffold output file with editable threshold values is provided as part of the iProX dataset (PXD037410).

### 2.10. Bioinformatic Analysis of Proteomic Sequence Data

Peptide and peptide-spectral-match (PSM) counts were quantified per protein according to the identifications from the Mascot/Scaffold pipeline described above. Proteins in each sample were intersected to identify proteins only detected in the adult female E/S, the adult male E/S, or both adult female and male E/S. The newly annotated *A. ceylanicum* proteins were assigned functional annotations using results from InterProScan v5.42 [40] to identify gene ontology [47] classifications and InterPro functional domains [48], and GhostKOALA v2.2 [49] to assign KEGG [50] annotations. Potentially secreted proteins were identified using both SignalP v5.0 [51] for signal peptides and transmembrane domains, and SecretomeP v2.0 [52] to identify proteins with non-classical secretion sequences (where any proteins with 2 or more transmembrane domains were not classified as secreted). Additional protein naming was performed using PANNZER [53] and Sma3s [54]. Significant functional enrichment for GO terms was performed using GOSTATS v2.50 [55] and for Interpro domains and KEGG pathways using WebGestalt v2019 [56] (FDR-adjusted *p* ≤ 0.05, minimum 3 genes differentially expressed, for all enrichment testing).

Protein conservation data across nematodes and hosts were quantified using BLAST [57] and OrthoFinder [58]. Protein sequence data were downloaded from WormBase Parasite [38] (WBPS16) for *A. duodenale* (PRJNA72581) and *N. brasiliensis* (PRJEB511). The *N. americanus* proteins were based on the improved annotation of the genome (PRJNA72135) presented in Logan et al., 2020 [6], and available for download on Nematode.net [43,44]. The *A. caninum* protein annotation was based on [59]. In addition, *Homo sapiens* (Human; GRCh38.103) was downloaded from Ensembl [60]. For BLAST comparisons, the top BLAST [57] hit for each *A. ceylanicum* protein was identified, including the E value, alignment length, % identity, and whether the top hit was reciprocal (NCBI blastp v2.7.1+, default settings). Orthofinder [58] (v2.4.1) was used to compare protein sequences across all species and define orthologous protein families (OPFs), using default values. E/S proteins identified in *N. americanus* were retrieved from Logan et al., 2020 [6] and aligned to all *A. ceylanicum* proteins using the BLAST results.

## 3. Results and Discussion

### 3.1. Improved Genome Annotation Identified ~10% More Peptides in Male and Female Hookworms

As a first step in the analysis, genome annotation improvement was performed to ensure the best possible and most complete characterization of the ES proteome. We re-annotated the genome of *A. ceylanicum* [29] using an improved gene calling pipeline utilizing RNA-seq data [39] and including more extensive up-to-date homologous protein databases (Table 1). The total number of predicted protein-coding genes decreased by 17,904 from 36,687 to 18,783 with substantial increases in both the mean exon and intron lengths. The mean coding sequence (CDS) length increased by 393 bp with the mean gene length (including introns and UTRs) more than doubling from 3.8 kb to 10 kb. The percentage of BUSCOs [42] detected among the predicted genes increased from 92.5% to 96% (with a 72% reduction in the number of fragmented BUSCOs).

Across both samples, 700 of the peptide sequences (7.94% of the 8818 total peptides) detected using the improved annotation were not part of the previous genome/proteome annotation for *A. ceylanicum* [29]. These peptides represented 10.8% and 9.7% of the total detected spectra in the adult female and male ESPs samples, respectively.

### 3.2. Female and Male Hookworm Secrete Similar Number of Proteins in Culture

**Figure 1:**
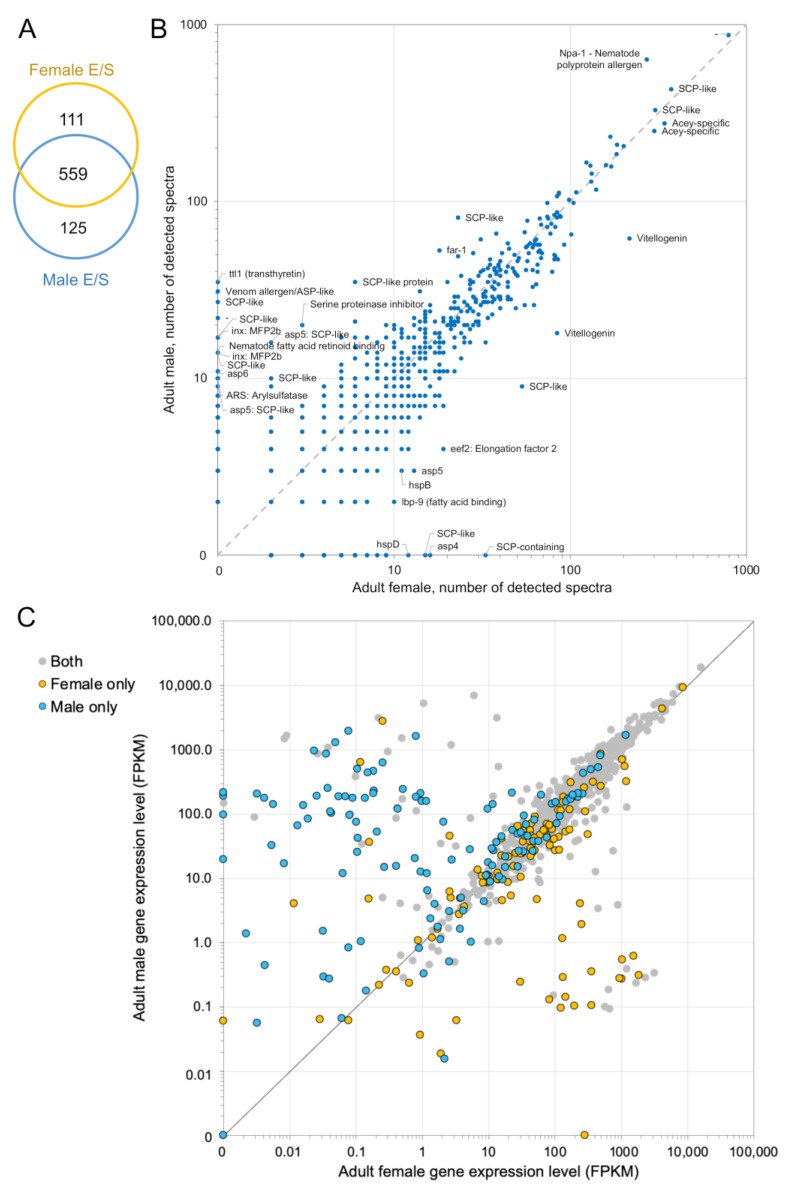
Distribution of ESPs proteins among female and male *A. ceylanicum*. (**A**) A Venn diagram showing the number of detected proteins in the ESPs of female and male *A. ceylanicum*. (**B**) A comparison of the relative abundance of proteins in the female and male samples, quantified by total number of spectra. Sex-specific ESPs are positioned along the X and Y axes. Brief functional annotations are provided for proteins with high sex specificity, and high overall abundance in both samples. (**C**) The corresponding adult-stage mRNA level for each of the ESPs in female and male *A. ceylanicum* [45].

Applying ultra-high-performance mass spectrometry methods using a hybrid quadrupole Orbitrap LC-MS system, a total of 795 unique proteins were detected (from 20.79 µg of female and 8.41 µg proteins in the male ESPs), with no peptides identified in the media-only control sample. Peptides and spectral counts from *A. ceylanicum* ESPs proteomics samples were identified using a Mascot/Scaffold pipeline (1% FDR peptide threshold, 95% protein confidence threshold, minimum 2 peptides per protein). Female and male hookworm secrete a similar number of ESPs in culture (670 vs. 684, respectively). Some 29.7% (*n* = 236) of all detected proteins were uniquely identified in female and male ESPs (Figure 1A). The relative abundance of all detected proteins in the adult male and adult female is shown for the two samples in Figure 1B, with most proteins having relatively similar abundance in both male and female ESPs. Figure 1C shows the adult-stage normalized mRNA levels (RNA-seq FPKM values) in female and male *A. ceylanicum* based on results from our previous study [45]. The female-specific ES proteins (orange) also have substantially higher mRNA levels in the female vs. male (below the diagonal). Likewise, many of the male-specific ES proteins (blue) also have substantially higher mRNA levels in the males vs. females (above the diagonal). This analysis shows that the sex-specific ESPs also show sex-specific mRNA levels, which supports their identification using an independent dataset.

### 3.3. Conserved ESPs in Female and Male A. Ceylanicum

A total of 559 proteins were detected in both female and male hookworm (Supplementary Table S1). Table 2 describes the 20 most abundant proteins in both female and male *A. ceylanicum*. As expected for ESPs, the proteins detected in both females and males were significantly enriched for signal peptides for secretion (63.9% of detected ESPs, vs. 18.6% for all proteins; *p* < 10^−10^, binomial distribution test, MS Excel; the threshold for significance *p* ≤ 0.01). They were also enriched for CAP domains (22.2% of proteins vs. 1.9% for all proteins; *p* < 10^−10^), which belong to sperm-coating protein (SCP)-like proteins, also known as SCP/Tpx-1/Ag5/PR-1/Sc7 domain-containing proteins (SCP/TAPS). This SCP-like family of proteins is highly expressed by parasitic nematodes and trematodes [61]. The major roles of SCP-like proteins are still unclear, but in hookworms, they have been implicated in larval skin penetration, the transition from the free-living to infectious stages [23,62,63], and host immune response modulation [64,65]. There was significant overlap in detected ESPs with orthologs of the ESPs detected in *N. americanus* in the previous study [6] (9.3% of detected ESPs, vs. 0.46% of all proteins; *p* < 10^−10^, binomial distribution test, MS Excel). Signal peptides, CAP domains, and ESPs are indicated for each protein in Table 2 and Supplementary Tables S1 and S2.

Of the sex-agnostic ESPs, ACEY_07898-1 (Acey_s0073.g740) is the most abundant by far, having more than 1.8 times as many total spectra assigned as the second-most abundant ESPs. Acey_s0073.g740 is a very large protein (3373 amino acids) with conservation only in other hookworm species, so there are no *C. elegans* or other annotated orthologs that can be used for comparison. The reciprocal best BLAST hit in *Necator americanus* (NAME_09181) was also by far the most abundant ESPs in the Logan et al. study [6], which provides confidence in its annotation and detection in the ESPs in this study and makes it an interesting candidate for future study. Among the most abundant proteins on the top list are also several implicated in immunomodulation, including ACEY_17598-1: Nematode polyprotein allergen Npa-1 [66], which was more than two-fold more abundant in the male than the female ESPs, and ACEY_09343-1: SCP-like protein [67], and which had very high mRNA levels in the RNA-seq dataset (Table 2). Npa-1, more commonly known as ABA-1, is a common nematode polyprotein allergen/antigen with a fatty acid binding property [68,69,70]. Ordinarily, nematodes do not have the potential to synthesize fatty acids and must obtain them from the host environment, and Npa-1 is likely used to meet this demand [71]. In the disease model, Npa-1 has been widely used as a serological marker for nematode infection, especially in diseases caused by *Ascaris lumbricoides* and *Ascaris suum* [72,73]. Npa-1 is abundant in the somatic tissues of the parasitic nematodes and is also discharged into the tissues of the host by the infective stages of nematode parasites [74].

KEGG pathway, gene ontology (GO) and Interpro domain enrichment among the proteins in both female and male (Table 3) indicated very strong enrichment for peptidases across all three enrichment tests, especially cysteine peptidases, as indicated by Interpro and GO. Cysteine peptidases are known to be particularly important for maintaining parasitic helminth survival in the host, including roles in the invasion of host tissues and in molting and embryogenesis [75]. Very strongly enriched is also the “exosome” KEGG term (*p* < 10^−10^) in hookworms; secreted extracellular vesicles are known to interact with host cells and induce immunomodulatory effects, through the action of both proteins and miRNAs [76].

Among the abundant proteins in both female and male *A. ceylanicum* ESPs, the most differentially abundant protein in female (216 spectra vs. 62 in males) is ACEY_08810-1: Vitellogenin (VIT) (Figure 1B). Vitellogenins are large glycolipoproteins representing the most conserved family of yolk proteins in oviparous species, where the proteins are naturally synthesized in extraovarian organs, transported to the circulatory system, and absorbed by the ovary [77,78]. Studies looking at the control of germline development and the role of soma-germline interactions in *C. elegans* revealed that vitellogenins, which serves as a nutrient source for the developing embryo, are made in the intestine, secreted into the somatic tissue, and taken up by oocytes through receptor-mediated endocytosis [79,80]. Vitellogenin, in addition to fatty acid and retinol-binding (FAR) proteins, are the main two lipid transport and storage proteins engaged in cross-membrane transport [81]. According to Wei et al. [81] more than three vitellogenins are fully expressed in the hookworm gut. Vitellogenin comprises four main domains including phosvitin, lipovitellin-1, lipovitellin-2, and a von Willebrand factor type D domain (vWFD) [82,83]. The three-dimensional structure of vitellogenin shows that lipovitellin and phosvitin domains form amphipathic structures of a lipid-binding pocket to accept their lipid cargo [84].

### 3.4. Proteins Detected Only in Female ESPs

Similar to the ESPs detected in both sexes, the 111 female-specific ESPs (14.6% of all female detected proteins) were enriched for signal peptides (49.5% of female-specific ESPs, vs. 18.6% for all proteins; *p* < 10^−10^, binomial distribution test, MS Excel) and CAP domains (9.0% vs. 1.9%; *p* = 8.7 × 10^−6^). This included SCP domain-containing protein (ACEY_06131-2) and Heat shock protein 90 (ACEY_06074-1) (Table 4). In previous research, many SCP-like proteins that were abundant in the ESPs of the iL3 stage of the canine hookworm *A. caninum* were considered “*Ancylostoma*-secreted proteins” [23,63] (ASPs), and many ASP proteins are identified as being only in the female ESPs here, including the second-most abundant protein, asp4. The most abundant female-specific ESPs also include heat shock proteins (Hsp), which act as molecular chaperones essential for regulating the folding and stability of different proteins during periods of increased temperature and other stress [85]. In *A. ceylanicum* female ES, only two GO terms were enriched among the 111 proteins detected. (Table 5). The represented GO terms related to molecular functions were related to “binding”, including pyridoxal phosphate binding (GO: 0030170) and vitamin B6 binding (GO: 0070279) (Table 5). Hookworm infection can induce vitamin B6 deficiency [86], and another parasitic helminth, *Schistosoma mansoni*, is known to acquire and metabolize host vitamin B6 during some life cycle stages [87], so these results are suggestive of a similar mechanism in *A. ceylanicum* adult females.

### 3.5. Proteins Detected Only in Male Hookworm

Similarly to the ESPs detected in both sexes and the female-specific ESPs, the 125 male-specific ESPs in *A. ceylanicum* (18.3% of all male-detected proteins) were significantly enriched for signal peptides (53.6% of male-specific ESPs, vs. 18.6% for all proteins; *p* < 10^−10^, binomial distribution test, MS Excel) and CAP domains (16.0% vs. 1.9%; *p* < 10^−10^). The most abundant male-specific ESPs was ACEY_02196-1, annotated as ttl1: precursor transthyretin-like protein 1 (Table 6). In a previous study, across 20 nematode secretomes, transthyretin proteins were the only component common across all species in the adult stage [88], highlighting an important yet unknown molecular function for these proteins. In mammals, transthyretin transports the hormones thyroxine and retinol into the circulation [89].

To further understand the functions of proteins identified in the ESPs of adult male *A. ceylanicum*, GO, KEGG, and Interpro domain functional enrichment was performed. In *A. ceylanicum* male ES, only 1 KEGG term was enriched among the 125 proteins detected, whereas 7 GO terms and 11 Interpro IDs were enriched (Table 7). Interestingly, the only male-specific KEGG-enriched term, lectin (4091) has been previously characterized for its importance in the reproductive physiology of *A. ceylanicum* [90]. Lectins are a single or multi-domain large family of calcium-dependent receptors with a wide variety of carbohydrate-recognition domains [91,92,93]. Our Interpro functional analysis predicted three C-type lectin domains-containing proteins including C-type lectin-like/link domain superfamily (IPR016186), C-type lectin fold (IPR016187), and C-type lectin-like (IPR001304) (Table 7). This lectin domain comprises about 110 to 130 residues. There is increasing evidence that these C-type lectins play an essential role in modulating helminth-mediated immune responses [91]. While these represent a set of male-specific C-type lectins, four others were detected in both males and females, and one was detected only in females. Similarly to the ESPs in both the females and males, the seven enriched GO terms among the male-specific ESPs were all related to peptidase activity (Table 7).

## 4. Conclusions

Here we report the exclusive sensitivity of an efficient mass spectrometry strategy combined with improved genome annotation of *A. ceylanicum* to identify secreted proteins in the ESPs of the adult female and male parasites. This is the first comprehensive profiling of the ESPs proteomes of female and male *A. ceylanicum*. The protein composition of profiled ESPs in adult *A. ceylanicum* leads to the conclusion that adult females and males secrete similar numbers of proteins into culture media, many of which are shared between the sexes, and a subset being differentially represented among the sexes, and related to sex-specific biology. This study increases our understanding of *A. ceylanicum* biology. Our results offer new leads for the development of vaccines and drugs to control the parasites, as well as the identification of new biologics that can be explored for their anti-inflammatory properties. The identified sex-specific secretomes of *A. ceylanicum* will facilitate other analyses, such as the enrichment of regulatory elements and motifs that mediate the expression of matching genes, information that should facilitate the engineering of transgenic *A. ceylanicum* to understand functions of specific genes, or to over-express different proteins of interest.

## Figures and Tables

**Table 1:** A comparison of genome annotation statistics, before and after re-annotation.

Statistic	Original Annotation (Schwarz et al., 2015) [29]	Re-Annotation
Number of genes	36,687	18,783
Number of mRNAs	65,583	22,928
Overlapping genes	3714	2170
Contained genes	1421	440
Mean gene length (bp)	3819	10,069
Mean exon length (bp)	138	246
Mean intron length (bp)	647	809
Mean CDS length (bp)	923	1316
Mean mRNAs per gene	1.8	1.2
Mean exons per mRNA	6.7	11.5
Complete BUSCOs	86.9%	94.5%
Fragmented BUSCOs	5.5%	1.5%
Missing BUSCOs	7.5%	4.0%

**Table 2:** The top twenty most abundant proteins in both female and male *A. ceylanicum*. Detected in Logan et al., 2020 [6]. Y = the top *N. americanus* BLAST hit is an ES product in that dataset; “-” = the top *N. americanus* BLAST hit is not an ES product in that dataset; “(N/A)” = there is no significant (E < 10^−5^) BLAST hit in the *N. americanus* protein set. Normalized mRNA levels (Fragments per kilobase per million reads, FPKM) based on the RNA-seq dataset from Bernot et al., 2020 [45].

Protein ID	Best Annotation	Conservation	Detected in Logan et al., 2020 [6]	Sig. pep.	CAP Domain	Adult E/S Proteomics				Avg. mRNA Level (FPKM)	
						Peptide Count		Spectral Count			
						F	M	F	M	Ad F	Ad M
ACEY_07898-1	Acey_s0073.g740	Clade V-specific	Y	-	-	241	261	788	874	638.1	486.6
ACEY_17598-1	5H561: Npa-1-Nematode polyprotein allergen	Clade V conserved (no human)	Y	Y	-	94	128	271	637	302.0	217.0
ACEY_09343-1	ANCCEY_02418: SCP-like protein	*Ancylostoma*-specific	Y	Y	Y	40	44	374	432	1622.5	3254.1
ACEY_06577-1	ASP2: SCP-like protein	Conserved in humans	Y	Y	Y	48	47	302	328	1876.3	2123.7
ACEY_15233-3	*A. ceylanicum*-specific	*A. ceylanicum*-specific	(N/A)	Y	-	19	19	343	277	126.8	140.3
ACEY_15233-2	*A. ceylanicum*-specific	*A. ceylanicum*-specific	(N/A)	Y	-	20	17	299	250	126.8	140.3
ACEY_02017-1	Acey_s0009.g591	*Ancylostoma*-specific	(N/A)	Y	-	18	13	200	206	4718.3	4053.5
ACEY_18401-1	IPR001283: Cysteine-rich secretory protein	*Ancylostoma*-specific	-	Y	Y	33	39	169	232	857.2	955.1
ACEY_07544-1	asp6: SCP-like protein	Clade V-specific	Y	Y	Y	27	28	184	210	1552.1	1634.6
ACEY_02018-1	Acey_s0009.g591	*Ancylostoma*-specific	(N/A)	-	-	18	15	183	186	2183.4	2253.6
ACEY_17268-1	SCP domain-containing protein	*Ancylostoma*-specific	(N/A)	Y	Y	18	22	170	158	16,104	18,856
ACEY_09344-1	asp6: SCP-like protein	*Ancylostoma*-specific	Y	Y	Y	34	38	159	161	1072.0	1697.4
ACEY_07899-1	Acey_s0073.g746	Clade V-specific	Y	-	-	48	57	130	160	352.1	276.2
ACEY_07543-1	asp6: Secreted protein 6	Clade V-specific	Y	Y	Y	31	35	123	166	449.4	657.6
ACEY_08810-1	VIT: Vitellogenin	Clade V conserved (no human)	Y	-	-	121	53	216	62	3155.6	0.3
ACEY_01671-3	asp5: SCP-like protein	Clade V conserved (no human)	Y	Y	Y	35	36	132	144	1053.7	1217.8
ACEY_13362-1	mtp: Metalloendopeptidase	*Ancylostoma*-specific	-	Y	-	27	23	131	130	213.3	380.1
ACEY_07900-1	Acey_s0073.g749	Clade V-specific	Y	-	-	63	62	140	117	136.1	162.5
ACEY_09375-1	IPR035109: Ancylostoma-associated secreted protein	*Ancylostoma*-specific	(N/A)	-	-	8	9	108	113	2634.1	2577.3
ACEY_02245-1	IPR035109: Ancylostoma-associated secreted protein	*Ancylostoma*-specific	(N/A)	Y	-	23	25	104	98	3281.0	3512.8

**Table 3:** Significantly enriched KEGG pathways, Gene Ontology (molecular function) terms, and Interpro domains among the 559 proteins detected in both male and female ESPs. The top 10 enriched terms are shown for each enrichment category.

Term	Description	Total Term Size	Number of Sig. Genes	FDR-Adjusted *p* Value
KEGG				
4147	Exosome	578	59	0
1002	Peptidases and inhibitors	406	53	0
3110	Chaperones and folding catalysts	206	32	0
4210	Apoptosis	108	26	0
4612	Antigen processing and presentation	73	23	0
4621	NOD-like receptor signaling pathway	86	21	6.3 × 10^−14^
536	Glycosaminoglycan binding proteins	126	24	1.9 × 10^−13^
4142	Lysosome	224	30	1.1 × 10^−12^
4140	Autophagy-animal	137	24	1.1 × 10^−12^
10	Glycolysis/Gluconeogenesis	62	10	8.9 × 10^−5^
GO molecular function				
GO: 0008233	peptidase activity	531	68	2.1 × 10^−24^
GO: 0016787	hydrolase activity	1238	92	4.5 × 10^−17^
GO: 0043169	cation binding	733	61	1.5 × 10^−12^
GO: 0046872	metal ion binding	727	60	2.9 × 10^−12^
GO: 0004175	endopeptidase activity	358	40	5.2 × 10^−12^
GO: 0004222	metalloendopeptidase activity	204	30	5.2 × 10^−12^
GO: 0008234	cysteine-type peptidase activity	105	22	5.3 × 10^−12^
GO: 0140096	catalytic activity, acting on a protein	1136	76	1.9 × 10^−11^
GO: 0008237	metallopeptidase activity	248	32	2.1 × 10^−11^
GO: 0046914	transition metal ion binding	468	40	1.3 × 10^−8^
Interpro domains				
IPR035940	CAP superfamily	465	136	0
IPR014044	CAP domain	394	124	0
IPR001283	Cysteine-rich secretory protein-related	306	94	0
IPR000668	Peptidase C1A, papain C-terminal	61	23	4.7 × 10^−14^
IPR025660	Cysteine peptidase, histidine active site	47	20	2.6 × 10^−13^
IPR000169	Cysteine peptidase, cysteine active site	52	20	2.2 × 10^−12^
IPR035109	Ancylostoma-associated secreted protein related	136	27	3.0 × 10^−9^
IPR025661	Cysteine peptidase, asparagine active site	39	15	4.1 × 10^−9^
IPR038765	Papain-like cysteine peptidase superfamily	110	23	2.6 × 10^−8^
IPR001506	Peptidase M12A	123	24	4.1 × 10^−8^

**Table 4:** The top 20 most abundant proteins that are only detected in the female ESPs samples. Detected in Logan et al., 2020 [6]. Y = the top *N. americanus* BLAST hit is an ES product in that dataset; “-” = the top *N. americanus* BLAST hit is not an ES product in that dataset; “(N/A)” = there is no significant (E < 10^−5^) BLAST hit in the *N. americanus* protein set. Normalized mRNA levels (Fragments per kilobase per million reads, FPKM) based on RNA-seq dataset from Bernot et al., 2020 [45].

Protein ID	Best Annotation	Conservation	Detected in Logan et al., 2020 [6]	Sig. pep.	CAP Domain	Adult E/S Proteomics				Avg. mRNA Level (FPKM)	
						Peptide Count		Spectral Count			
						F	M	F	M	Ad F	Ad M
ACEY_06131-2	SCP domain-containing protein	*Ancylostoma*-specific	Y	Y	Y	14	0	33	0	15.7	41.7
ACEY_11619-1	asp4: Secreted protein 4	*Ancylostoma*-specific	-	Y	Y	7	0	16	0	197.3	0.10
ACEY_18091-1	ANCCEY_04696: SCP-like protein	*Ancylostoma*-specific	Y	-	Y	6	0	15	0	0.03	0.06
ACEY_06074-1	hspD: Heat shock protein 90	Conserved in humans	-	-	-	11	0	12	0	1143.4	544.7
ACEY_00553-1	nsbp: Putative nucleosome binding protein	Conserved in humans	-	-	-	9	0	9	0	1037.1	701.5
ACEY_03322-1	IPR014044:CAP domain	*A. ceylanicum*-specific	Y	Y	Y	8	0	9	0	0.16	36.2
ACEY_06771-1	HGS: Metalloendopeptidase	*A. ceylanicum*-specific	-	Y	-	8	0	9	0	1852.0	0.31
ACEY_17074-1	IPR035109: *Ancylostoma*-associated secreted protein	*A. ceylanicum*-specific	(N/A)	-	-	8	0	9	0	49.0	42.3
ACEY_06945-1	ttn-1: Immunoglobulin I-set domain protein	Conserved in humans	-	-	-	8	0	8	0	0.29	0.37
ACEY_03325-1	IPR002172:Low-density lipoprotein (LDL) receptor	Clade V-specific	(N/A)	Y	-	7	0	8	0	0.26	2796.7
ACEY_16559-1	r2: Ribonucleoside-diphosphate reductase	Conserved in humans	-	-	-	6	0	7	0	315	48
ACEY_16507-1	CBG16650: Deoxyribonuclease II	Clade V conserved (no human)	-	-	-	5	0	7	0	44.5	70.7
ACEY_02929-1	CBG06778: Trypsin Inhibitor like cysteine rich domain	Clade V conserved (no human)	-	Y	-	6	0	6	0	129.9	1.2
ACEY_08051-1	Acey_s0076.g1061	Clade V-specific	-	Y	-	6	0	6	0	146.0	0.14
ACEY_14605-1	hspa4: Heat shock protein 105 kDa	Conserved in humans	-	-	-	6	0	6	0	23.8	15.3
ACEY_04573-1	-	Clade V conserved (no human)	Y	Y	-	5	0	6	0	0.12	632.5
ACEY_15954-1	IPR035109: *Ancylostoma*-associated secreted protein	Clade V-specific	-	Y	-	5	0	6	0	32.9	54.5
ACEY_00191-1	GOT2: Aspartate aminotransferase, mitochondrial	Conserved in humans	-	-	-	5	0	6	0	169.2	56.4
ACEY_03760-1	lgmn: Legumain	Conserved in humans	-	Y	-	5	0	6	0	279.9	256.6
ACEY_10061-1	spna2: Spectrin alpha chain	Conserved in humans	-	-	-	5	0	6	0	31.0	24.2

**Table 5:** Gene Ontology (GO) term analysis, and molecular function in adult female ESPs of *A. ceylanicum*.

Term	Description	Total Term Size	Number of Sig. Genes	FDR-Adjusted *p* Value
GO molecular function				
GO: 0030170	pyridoxal phosphate binding	35	3	0.037
GO: 0070279	vitamin B6 binding	35	3	0.037

**Table 6:** The top twenty most abundant proteins that are only detected in the male samples. Detected in Logan et al., 2020 [6]. Y = the top *N. americanus* BLAST hit is an ES product in that dataset; “-” = the top *N. americanus* BLAST hit is not an ES product in that dataset; “(N/A)” = there is no significant (E < 10^−5^) BLAST hit in the *N. americanus* protein set. Normalized mRNA levels (Fragments per kilobase per million reads, FPKM) based on the RNA-seq dataset from Bernot et al., 2020 [45].

Protein ID	Best Annotation	Conservation	Detected in Logan et al., 2020 [6]	Sig. pep.	CAP Domain	Adult E/S Proteomics				Avg. mRNA Level (FPKM)	
						Peptide Count		Spectral Count			
						F	M	F	M	Ad F	Ad M
ACEY_02196-1	ttl1: Precursor transthyretin like protein 1	Clade V conserved (no human)	-	Y	-	0	7	0	35	354.7	490.3
ACEY_13694-1	Venom allergen/*Ancylostoma* secreted protein-like 18	*Ancylostoma*-specific	-	Y	Y	0	6	0	31	223.7	184.5
ACEY_06112-1	NECAME_07497: SCP-like protein	*Ancylostoma*-specific	Y	Y	Y	0	8	0	27	0.21	52.4
ACEY_07390-1	Acey_s0065.g3604	Clade V-specific	-	Y	-	0	17	0	22	0.94	12.7
ACEY_15398-1	inx: MFP2b	Clade V conserved (no human)	-	-	-	0	13	0	17	0.14	177.2
ACEY_06326-1	Acey_s0048.g1585: SCP-like protein	Clade V-specific	-	Y	Y	0	10	0	17	9.95	17.6
ACEY_01267-1	Nematode fatty acid retinoid binding protein	*Ancylostoma*-specific	Y	-	-	0	14	0	14	0.80	1623.7
ACEY_15396-1	inx: MFP2b	Clade V conserved (no human)	-	-	-	0	12	0	14	0.080	95.5
ACEY_08562-1	Acey_s0087.g2034: SCP-like protein	*Ancylostoma*-specific	(N/A)	Y	Y	0	9	0	14	0.04	852.7
ACEY_16228-1	SCP domain-containing protein	*A. ceylanicum*-specific	Y	-	Y	0	4	0	14	2.58	3.0
ACEY_13234-1	von Willebrand factor domain containing protein	Clade V conserved (no human)	-	-	-	0	9	0	11	1.2	12
ACEY_01957-1	asp6: SCP-like protein	*Ancylostoma*-specific	Y	-	Y	0	8	0	11	0.12	1.0
ACEY_09376-1	ARS: Arylsulfatase	Conserved in humans	-	Y	-	0	9	0	10	2.1	74.7
ACEY_00325-1	asp5: SCP-like protein	*Ancylostoma*-specific	Y	Y	Y	0	7	0	10	1.0	158.7
ACEY_02166-1	Acey_s0010.g910	Clade V-specific	-	Y	-	0	8	0	9	0.10	498.8
ACEY_15765-1	*Ancylostoma*-specific	*Ancylostoma*-specific	(N/A)	Y	-	0	7	0	9	0.43	119.5
ACEY_02233-1	ANCCEY_04287: SCP-like protein	Clade V-specific	Y	Y	Y	0	7	0	8	0.51	241.0
ACEY_15766-1	Acey_s0439.g1485	*Ancylostoma*-specific	(N/A)	Y	-	0	6	0	8	11.7	141.1
ACEY_02212-1	asp6: SCP-like protein	*Ancylostoma*-specific	Y	Y	Y	0	6	0	8	0.032	0.29
ACEY_07632-1	PPN: Kunitz/Bovine pancreatic trypsin inhibitor	*Ancylostoma*-specific	-	Y	-	0	6	0	8	0.038	252.7

**Table 7:** KEGG, GO, and Interpro analysis of molecular function and domain functional annotations in adult male ESPs of *A. ceylanicum*.

Term	Description	Total Term Size	Number of Sig. Genes	FDR-Adjusted *p* Value
KEGG				
4091	Lectins	43	4	3.7 × 10^−2^
GO molecular function				
GO: 0061135	endopeptidase regulator activity	146	13	1.8 × 10^−10^
GO: 0004866	endopeptidase inhibitor activity	146	13	1.8 × 10^−10^
GO: 0030414	peptidase inhibitor activity	152	13	2.0 × 10^−10^
GO: 0061134	peptidase regulator activity	156	13	2.1 × 10^−10^
GO: 0004867	serine-type endopeptidase inhibitor activity	130	12	3.4 × 10^−10^
GO: 0030234	enzyme regulator activity	273	14	1.4 × 10^−8^
GO: 0098772	molecular function regulator	298	14	3.7 × 10^−8^
Interpro domains				
IPR035940	CAP superfamily	465	22	3.2 × 10^−7^
IPR014044	CAP domain	394	20	3.5 × 10^−7^
IPR021010	Cytosolic motility protein	7	5	7.2 × 10^−7^
IPR036880	Pancreatic trypsin inhibitor Kunitz domain superfamily	112	11	2.6 × 10^−6^
IPR002223	Pancreatic trypsin inhibitor Kunitz domain	107	10	1.7 × 10^−5^
IPR001283	Cysteine-rich secretory protein-related	306	15	3.6 × 10^−5^
IPR020901	Proteinase inhibitor I2, Kunitz, conserved site	76	8	1.1 × 10^−4^
IPR016186	C-type lectin-like/link domain superfamily	111	9	1.7 × 10^−4^
IPR016187	C-type lectin fold	117	9	2.4 × 10^−4^
IPR001304	C-type lectin-like	102	8	7.5 × 10^−4^
IPR008962	PapD-like superfamily	56	5	0.025

## Data Availability

The mass spectrometry proteomics data have been deposited to the ProteomeXchange Consortium (http://proteomecentral.proteomexchange.org, accessed on 1 November 2022) via the iProX partner repository with the dataset identifier PXD037410, under project ID IPX0005214000. The accessions for each sample are IPX0005214001 (Adult_Female_ESPs), IPX0005214002 (Adult_Male_ESPs) and IPX0005214003 (Media-only control).

## Supplementary Materials

The following supporting information can be downloaded at: https://www.mdpi.com/article/10.3390/pathogens12010095/s1, Table S1: Gene database including functional annotations (Sma3s, Pannzer2, KEGG, Interpro, Gene Ontology), conservation data, remapped relative mRNA levels (FPKM) for RNA-seq samples spanning the *A. ceylanicum* life cycle, and peptide and summed peptide spectral match (PSM) counts for all detected proteins in the ESPs samples. Table S2: Peptide spectral match (PSM) counts per detected peptide, according to output from Scaffold.
